# Omadacycline Gut Microbiome Exposure Does Not Induce Clostridium difficile Proliferation or Toxin Production in a Model That Simulates the Proximal, Medial, and Distal Human Colon

**DOI:** 10.1128/AAC.01581-18

**Published:** 2019-01-29

**Authors:** Ines B. Moura, Anthony M. Buckley, Duncan Ewin, Sharie Shearman, Emma Clark, Mark H. Wilcox, Caroline H. Chilton

**Affiliations:** a Leeds Institute of Biomedical and Clinical Sciences, Faculty of Medicine and Health, University of Leeds, Leeds, United Kingdom; b Department of Microbiology, Leeds Teaching Hospitals NHS Trust, The General Infirmary, Leeds, United Kingdom

**Keywords:** Clostridium difficile infection, omadacycline, gut microflora, gut model

## Abstract

A clinically reflective model of the human colon was used to investigate the effects of the broad-spectrum antibiotic omadacycline on the gut microbiome and the subsequent potential to induce simulated Clostridium difficile infection (CDI). Triple-stage chemostat gut models were inoculated with pooled human fecal slurry from healthy volunteers (age, ≥60 years). Models were challenged twice with 10^7^ CFU C. difficile spores (PCR ribotype 027). Omadacycline effects were assessed in a single gut model. Observations were confirmed in a parallel study with omadacycline and moxifloxacin. Antibiotic instillation was performed once daily for 7 days. The models were observed for 3 weeks postantibiotic challenge. Gut microbiota populations and C. difficile total viable and spore counts were enumerated daily by culture. Cytotoxin titers and antibiotic concentrations were also measured. Gut microbiota populations were stable before antibiotic challenge. Moxifloxacin instillation caused an ∼4 log_10_ CFU/ml decline in enterococci and Bacteroides fragilis group populations and an ∼3 log_10_ CFU/ml decline in bifidobacteria and lactobacilli, followed by simulated CDI (vegetative cell proliferation and detectable toxin). In both models, omadacycline instillation decreased populations of bifidobacteria (∼8 log_10_ CFU/ml), B. fragilis group populations (7 to 8 log_10_ CFU/ml), lactobacilli (2 to 6 log_10_ CFU/ml), and enterococci (4 to 6 log_10_ CFU/ml). Despite these microbial shifts, there was no evidence of C. difficile bacteria germination or toxin production. In contrast to moxifloxacin, omadacycline exposure did not facilitate simulated CDI, suggesting this antibiotic may have a low propensity to induce CDI in the clinical setting.

## Abstract (precis)

A clinically reflective model of the human colon was used to investigate the effects of the broad-spectrum antibiotic omadacycline on the gut microbiome and the subsequent potential to induce simulated Clostridium difficile infection (CDI). Triple-stage chemostat gut models were inoculated with pooled human fecal slurry from healthy volunteers (age, ≥60 years).

## INTRODUCTION

Clostridium difficile infection (CDI) is the leading cause of nosocomial antibiotic-associated diarrhea around the world and a major cause of morbidity in the hospitalized elderly (1).

A stable gut microbiota provides colonization resistance, a key factor in preventing C. difficile colonization and proliferation (2). Antibiotic use is a substantial risk factor for CDI due to the disruption of the microbiota. In particular, broad-spectrum antibiotics (e.g., fluoroquinolones, cephalosporins, and clindamycin) represent an increased CDI risk, especially when they are associated with the protracted impairment of microbiota populations (3, 4).

Omadacycline is a potent aminomethylcycline, with *in vitro* activity against Gram-positive and Gram-negative bacteria, including methicillin-susceptible and -resistant Staphylococcus aureus, coagulase-negative staphylococci, Enterococcus faecalis, Enterococcus faecium, Streptococcus pneumoniae, Klebsiella pneumoniae, and Bacteroides fragilis (5, 6). Omadacycline has recently completed phase 3 clinical trials for acute bacterial skin and skin structure infections and community-acquired bacterial pneumonia (7). Similar to other tetracyclines, omadacycline inhibits protein synthesis by binding to the 30S ribosomal subunit, although this novel antimicrobial has been structurally modified to overcome efflux mechanisms (6, 8). The effect of omadacycline on the normal gut microbiome and its subsequent potential for the induction of CDI have not been investigated.

The *in vitro* gut model has been previously used to study antibiotic predisposition to simulated CDI using epidemic virulent strains, and the results appear to correlate well with clinical CDI risk (9). Antibiotics known to have a high propensity to induce CDI clinically have induced CDI in this model (9–12). Conversely, piperacillin-tazobactam and tigecycline, antibiotics with a low propensity to induce CDI, did not promote C. difficile germination and toxin production in the gut model (13, 14).

This study investigated the effects of omadacycline instillation on the normal gut microbiome populations and the subsequent potential for induction of CDI compared with those of moxifloxacin.

## RESULTS

### Effects of omadacycline instillation on gut microflora and C. difficile populations.

**FIG 1:**
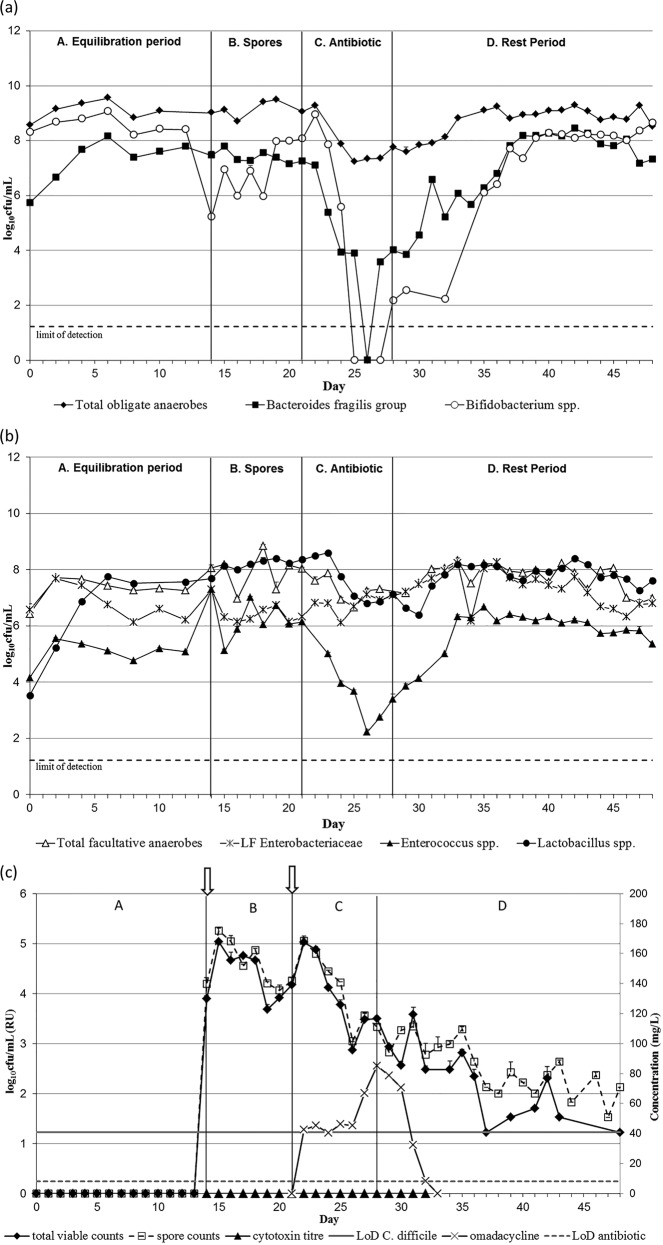
Mean obligate anaerobic gut microbiota populations (log10 CFU/ml) (a); mean facultative anaerobic gut microbiota populations (log10 CFU/ml) (b); and mean *C. difficile* total viable counts and spore counts (log10 CFU/ml), cytotoxin titers (relative units, RU), and antimicrobial concentration (mg/liter) (c) in vessel 3 of model OMC. All vertical arrows mark the addition of *C. difficile* spores to the model, and horizontal arrow marks the period of antibiotic instillation. LF *Enterobacteriaceae*, lactose-fermenting *Enterobacteriaceae*; LoD, limit of detection.

A single triple-stage chemostat model (see Fig. S1 in the supplemental material) containing a stable microbiota derived from healthy stool samples was used to investigate the effects of omadacycline on the colonic microflora and the propensity of the antibiotic to induce simulated CDI. This gut model, here referred to as OMC, was instilled with a clinically reflective regimen of omadacycline, and microbial populations, including Clostridium difficile total counts and spores, were monitored daily. The changes in gut microbial populations in vessels 2 and 3 were similar during omadacycline instillation. Vessel 3 is of most clinical relevance for CDI (see Materials and Methods). Results of bacterial enumeration in vessel 3 are shown in Fig. 1, whereas vessel 2 data are presented as supplemental material (see Fig. S2). Omadacycline instillation caused a decline of ∼7 log_10_ CFU/ml in B. fragilis group bacteria (standard error [SE], ±0.16) and a decline of ∼8 log_10_ CFU/ml in bifidobacteria populations (SE, ±0.14). Both of these bacterial populations decreased to below the limit of detection (∼1.2 log_10_ CFU/ml) in vessel 3 (Fig. 1a). Enterococcus spp. (SE, ±0.18) and lactobacilli (SE, ± 0.1) decreased ∼4 log_10_ CFU/ml and ∼2 log_10_ CFU/ml, respectively (Fig. 1b). Lactose-fermenting Enterobacteriaceae populations (SE, ±0.18) increased during omadacycline exposure, between 2 log_10_ CFU/ml in vessel 2 and 1 log_10_ CFU/ml in vessel 3. Overall, a 2-log decrease in total viable counts (SE, ±0.18) was observed following omadacycline exposure, whereas total facultative anaerobic populations (SE, ±0.25) remained stable. The recovery of gut microbiota populations was observed 7 days after omadacycline instillation ended, and populations had returned to steady-state levels by the end of the experiment.

C. difficile total viable counts (TVCs; SE, ±0.17) remained roughly equal to spore counts (SE, ±0.23; as distinguished by alcohol tolerance) throughout the experiment in all three vessels of OMC, indicating that all C. difficile bacteria remained as spores (vessel 3 data shown in Fig. 1c; for vessel 2 data, see Fig. S2c in the supplemental material). Vegetative cell proliferation was not observed, and toxin was not detected in any of the vessels. Simulated CDI did not occur in the OMC gut model.

In OMC, the mean bioactive omadacycline concentrations peaked at 384 (SE, ±64.8) mg/liter, 163 (SE, ±30.3) mg/liter, and 85 (SE, ±46.7) mg/liter in vessels 1, 2 and 3, respectively. Antimicrobial concentrations in OMC were detectable for 2, 3, and 5 days in vessels 1, 2, and 3, respectively, in the postantibiotic period.

### *In vitro* comparison of omadacycline and moxifloxacin propensity to induce CDI.

Following the observation that omadacycline did not induce simulated CDI in model OMC, a pair of gut models comparing omadacycline (OMC1) and moxifloxacin (MOX) exposure was run. Moxifloxacin has previously been shown to induce simulated CDI in the *in vitro* gut model (12) and has been linked to the spread of the epidemic 027 ribotype (15).

**FIG 2:**
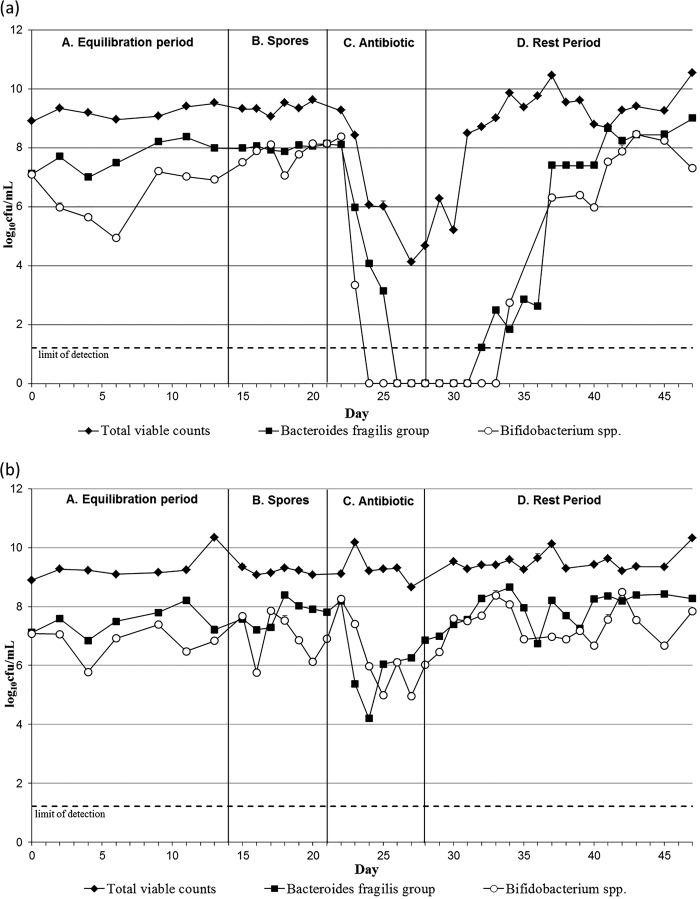
Mean obligate anaerobic gut microbiota populations (log_10_ CFU/ml), including standard error bars, in vessel 3 of model OMC1 (omadacycline dosing) (a) and model MOX (moxifloxacin dosing) (b).

**FIG 3:**
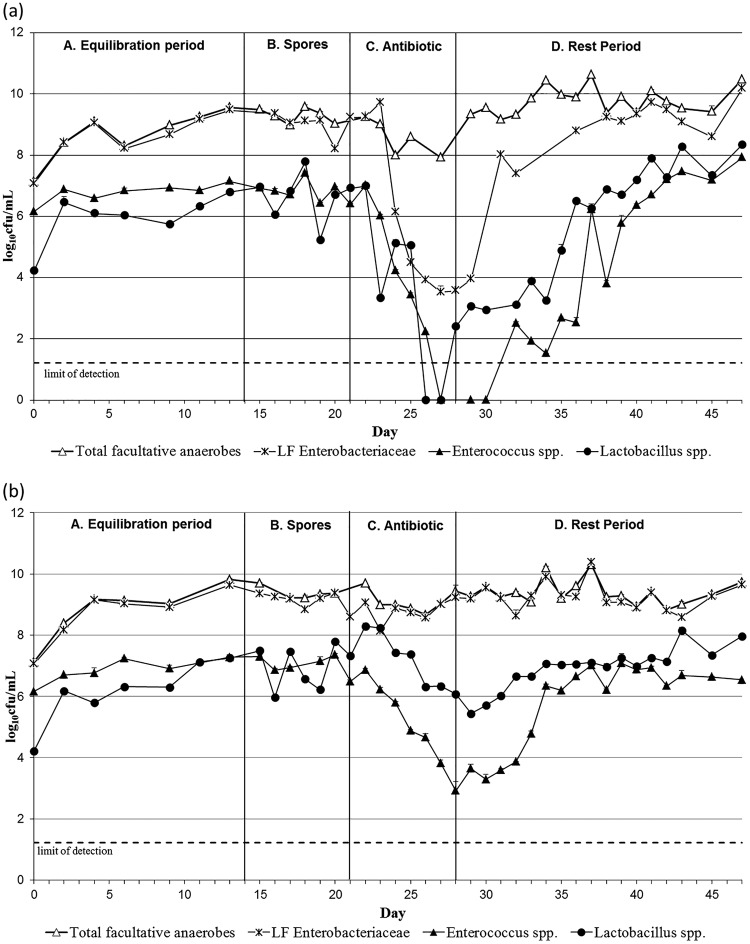
Mean facultative anaerobic gut microbiota populations (log_10_ CFU/ml), including standard error bars, in vessel 3 of model OMC1 (omadacycline dosing) (a) and model MOX (moxifloxacin dosing) (b). LF *Enterobacteriaceae*, lactose-fermenting *Enterobacteriaceae*.

Again, population changes in vessel 2 and vessel 3 were similar, so vessel 3 data only are shown in Fig. 2 and 3, whereas vessel 2 data are presented in Fig.S3 and S4 in the supplemental material. The addition of C. difficile spores to the gut models did not cause any variations in the intestinal bacterial populations monitored in OMC1 and MOX.

In OMC1, omadacycline exposure caused declines in B. fragilis group bacteria (SE, ±0.11) and bifidobacteria (SE, ±0.24) of ∼8 log_10_ CFU/ml, to below the limit of detection (Fig. 2a). Lactobacilli (SE, ±0.20) and Enterococcus spp. (SE, ±0.25) populations declined by ∼6 log_10_ CFU/ml (Fig. 3a), and lactose-fermenting Enterobacteriaceae populations (SE, ±0.099) decreased approximately ∼5 log_10_ CFU/ml. Overall, omadacycline instillation in OMC1 led to a decrease of ∼5 log_10_ CFU/ml in the total viable counts (SE, ±0.2), although the total facultative anaerobic populations (SE, ±0.18) remained fairly stable throughout the experiment, suggesting a decline of the obligate anaerobic populations. Gut microbiota had returned to steady-state levels by the end of the experiment, with the initial recoveries being observed 9 days after omadacycline instillation ended.

Moxifloxacin instillation caused marked declines in B. fragilis group populations (SE, ±0.1), between ∼4 log_10_ CFU/ml in vessel 3 (Fig. 2b) and ∼8 log_10_ CFU/ml in vessel 2 (see Fig. S3b). Populations of bifidobacteria (SE, ±0.18) and lactobacilli (SE, ±0.1) declined ∼3 log_10_ CFU/ml in both vessels 2 and 3 (Fig. 2b and 3b; see also Fig.S3b and S4b). Enterococci populations (SE, ±0.18) also decreased ∼4 log_10_ CFU/ml in both vessels. Lactose-fermenting Enterobacteriaceae populations (SE, ±0.18) remained stable in vessel 3 but declined ∼2 log_10_ CFU/ml in vessel 2 (Fig. 3b; see also Fig. S4b). Overall, both obligate (SE, ±0.14) and facultative anaerobes (SE, ±0.19) remained constant during and after moxifloxacin instillation, as suggested by the total viable counts. Microbiota populations returned to steady-state numbers approximately 1 week after moxifloxacin instillation ended (Fig. 2b and 3b).

### Effect of omadacycline and moxifloxacin on C. difficile.

**FIG 4:**
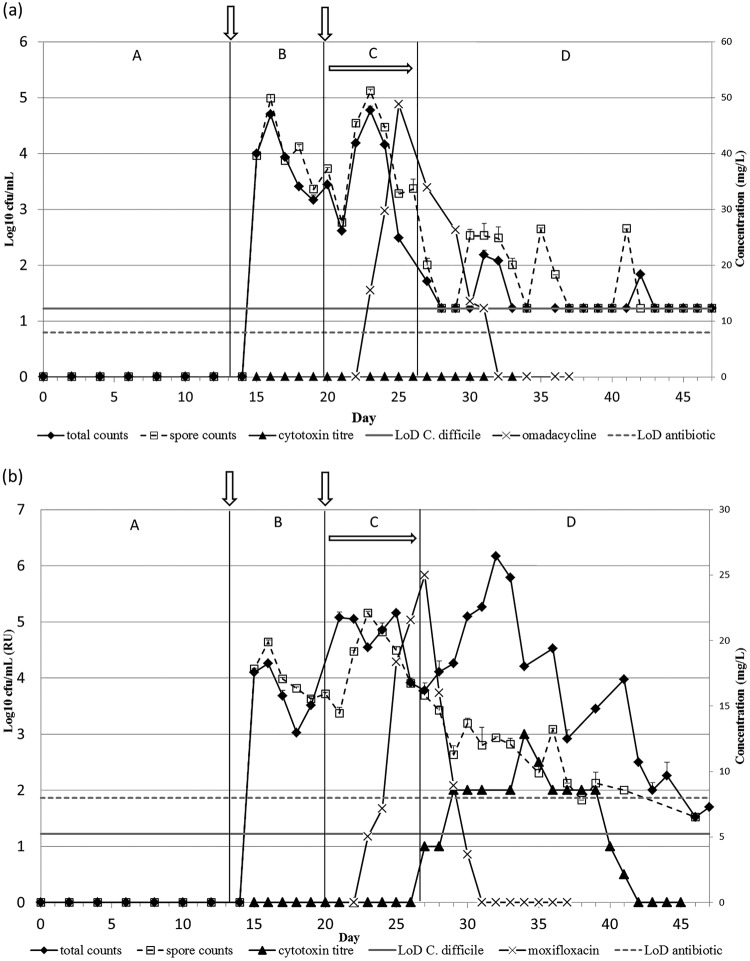
Mean *C. difficile* total viable counts and spore counts (log10 CFU/ml), cytotoxin titers (relative units, RU), and antimicrobial concentration (mg/liter) in vessel 3 of model OMC1 (omadacycline dosing) (a) and model MOX (moxifloxacin dosing) (b). Periods A to D are defined in Fig. 5. Vertical arrows mark the addition of *C. difficile* spores to the model, and horizontal arrow marks the period of antibiotic instillation. LoD, limit of detection.

As was observed in OMC, TVCs (SE, ±0.09) in OMC1 remained similar to spore counts (SE, ±0.17) throughout the experiment in all vessels. C. difficile vegetative cell proliferation was not observed, and toxin was not detected (Fig. 4a; see also Fig. S5a).

In all vessels of the MOX gut model, C. difficile remained as spores during the internal control stage (period B); however, during moxifloxacin instillation (period C), an increase in TVCs (SE, ±0.16) compared with spore counts (SE, ±0.16) was observed, corresponding to spore germination and vegetative cell proliferation. TVCs peaked at ∼4.2 log_10_ CFU/ml on day 30 in vessel 1 (data not shown) and ∼6 log_10_ CFU/ml in vessel 2 (see Fig. S5b) and vessel 3 at day 33 (Fig. 4b). The increase in TVCs was concomitant with the detection of C. difficile cytotoxin, which reached a peak titer of 2 relative units in vessel 1 and 3 relative units in vessels 2 and 3. Both TVCs and toxin titers decreased toward the end of the experiment, with toxin undetectable in all vessels by day 42.

### Antimicrobial concentrations in the gut models.

Omadacycline concentrations in OMC1 peaked at 242 (SE, ±41) mg/liter, 119 (SE, ±11.47) mg/liter, and 48 (SE, ±2.78) mg/liter in vessels 1, 2, and 3, respectively. Omadacycline remained detectable for 2 days in vessels 1 and 2 (vessel 2 data are shown in Fig. S5a) and for 3 days in vessel 3 (Fig. 4a) after antibiotic instillation ceased.

Moxifloxacin concentrations peaked at 55 (SE, ±5.2) mg/liter, 34 (SE, ±2.3) mg/liter, and 25 (SE, ±3.3) mg/liter in vessels 1, 2, and 3, respectively, and remained detectable for 2 days in the postantibiotic period in vessels 1 and 2 (vessel 2 data are shown in Fig. S5b), and for 3 days in vessel 3 (Fig. 4b).

## DISCUSSION

The instillation of omadacycline did not elicit simulated CDI in an *in vitro* triple-stage chemostat model (OMC). To further confirm OMC gut model observations, a second experiment was conducted, where an omadacycline gut model (OMC1) was run in parallel with a model dosed with moxifloxacin (MOX) in order to compare the potential of each antibiotic to induce simulated CDI. Despite the fact that gut microbiota dysbiosis appeared more severe following omadacycline exposure, C. difficile germination and toxin production (simulated CDI) were observed only in the model instilled with moxifloxacin. The use of different fecal emulsions to initiate OMC and OMC1 aimed to analyze the effect of the antimicrobial in unrelated human intestinal microbiota. Similar to expected variation in different individuals, variations in the extent of the microflora disruption were observed in OMC and OMC1; however, CDI was not observed in either of the models.

Omadacycline effects on the anaerobic gut microbiota populations were similar in OMC and OMC1, with all measured anaerobic populations affected, most notably bifidobacteria and B. fragilis group bacteria, which declined to below the limit of detection. The main differences between these models were observed during antimicrobial instillation (period C) in facultative anaerobic populations. An increase of ∼1 log_10_ CFU/ml in Enterobacteriaceae populations was observed in OMC, in opposition to the 5 log_10_ CFU/ml decline observed in OMC1. This variation is likely to be associated with the different fecal slurry used to initiate each model. A study investigating 8,345 clinical Enterobacteriaceae isolates has reported MICs to omadacycline ranging from ≤0.25mg/liter (4.1%) to ≥32mg/liter (3.4%) (16). Given the complexity of the normal microbiota, the presence of Enterobacteriaceae strains with a wide range of susceptibilities to omadacycline could explain the differences observed in these populations during antibiotic instillation in the gut model. Compared with omadacycline, moxifloxacin instillation caused a less pronounced decline in B. fragilis group bacteria, enterococci, and lactobacilli populations in vessels 2 and 3, but this was followed by the detection of toxin in all three vessels. These findings are consistent with previous data (12).

In a phase 1 clinical trial, omadacycline metabolism and recovery rates were investigated in healthy individuals, following the ingestion of the recommended daily oral dose of 300 mg of the antimicrobial (7, 17). Approximately 95% of the antimicrobial was excreted, predominantly through feces (mean, 81.1%) and urine (14.4%), without drug metabolites being detected. The highest concentration of omadacycline registered in human feces in that study was ∼430 mg/Kg (unpublished data), which informed the use of a 430 mg/liter once daily dosing regimen in this study. Peak omadacycline levels observed in vessel 3 were lower than 430 mg/liter, potentially suggesting the occurrence of drug metabolism or sequestration. The lack of metabolites observed in the feces clinically (17) indicates that sequestration into the biofilm is more likely. Observed omadacycline concentrations were higher than those determined during moxifloxacin dosing. Moxifloxacin concentrations observed in vessels 1, 2, and 3 were consistent with the values reported in previous moxifloxacin gut models (12) and are reflective of gut levels seen *in vivo* (18). Microbial recovery in either OMC model was observed between 7 and 9 days postcessation of omadacycline.

The observations in our triple-stage model have been shown to correlate with clinical observations of antimicrobial propensity to induce CDI *in vivo* (9–14) and have changed United Kingdom national antibiotic prescribing guidelines (19). Antibiotics, such as cephalosporins (9), clindamycin (10), co-amoxyclav (11), or fluoroquinolones (12), known to have a high propensity to induce CDI clinically, have induced CDI in this model. Similarly, piperacillin-tazobactam (13) and tigecycline (14), two antibiotics considered of low risk for CDI clinically, have not induced simulated CDI in the gut model. An intact colonic microbiota has an important role in protecting the organism against intestinal bacterial pathogens (2, 20). Broad-spectrum antibiotic therapy causes a decline of the commensal microflora, leading to a diminution of microbiota diversity and the subsequent opportunity for the outgrowth of pathogens, such as C. difficile (3, 4). Notably, the gut model studies that investigated piperacillin-tazobactam (13) and tigecycline (14) also observed a significant decrease in intestinal microflora populations and yet an absence of induction of CDI. The tigecycline MIC of the PCR ribotype 027 strain used in this study was 0.06 mg/liter, with tigecycline concentrations remaining above 1 mg/liter 5 days after cessation of antibiotic instillation (14).

In the present study, the omadacycline MIC (0.25 mg/liter) of the C. difficile strain added to the gut model was assessed by the recommended agar dilution method (21) (data not shown). C. difficile ATCC 700057 (MIC, 1 mg/liter) was used as a control (22). Omadacycline levels dropped to below the limit of detection (8 mg/liter) at day 34 and 32, in OMC and OMC1, respectively. While the bioactive concentration of omadacycline remains higher than the strain MIC, it would be expected that any C. difficile spore outgrowth would be prevented; however, once the concentrations decrease below the MIC, spore outgrowth and vegetative cell proliferation would no longer be prevented. This is observed following clindamycin exposure in the gut model. A C. difficile strain with a clindamycin MIC of 0.5 mg/liter does not germinate during clindamycin instillation (10), but CDI is consistently observed in the postantibiotic period. Once clindamycin levels fall below the limit of detection (typically around 5- to 7-days postinstillation), C. difficile vegetative proliferation and toxin are observed. It is possible that omadacycline, tigecycline, and piperacillin-tazobactam intrinsic activity against C. difficile persists long enough to prevent its proliferation, even when a potential niche has been created by antibiotic exposure. Baines et al. (13) have previously proposed that the biofilm formed on the walls of the vessels may contribute to the swift recovery of the bacterial populations in the gut model and operates as a bacterial reservoir during antimicrobial dosing. It is possible that the biofilm formed in the chemostat model can also contribute to the persistence of the drug to a concentration higher than 0.25 mg/liter, sufficient to inhibit C. difficile spore germination and, therefore, prevent simulated CDI in the gut model. The sequestration of fidaxomicin into the biofilm of the gut model has previously been observed and hypothesized to contribute to the prolonged detection of fidaxomicin in the gut model and patient stool (23). Moreover, the relatively rapid reconstitution of gut microflora populations after the cessation of antimicrobial instillation will provide further protection against CDI.

Despite the disruptive effects of omadacycline to the colonic microflora, compared with the present and previous gut model studies, our data suggest that omadacycline may have a lower-risk for CDI induction than moxifloxacin and other fluoroquinolones. These observations agree with published studies focusing on the impact of antibiotic exposure on CDI risk in hospital (24) and community settings (25), which reported tetracyclines to be among the classes of antimicrobials with the lowest risk for CDI induction (26). Furthermore, in a recent phase 3 community-acquired pneumonia clinical trial, there were no cases of CDI observed in patients treated with omadacycline, compared with eight cases (2%) in those who received moxifloxacin (27). Further human *in vivo* data are needed to confirm these observations suggesting a low CDI propensity for omadacycline.

## MATERIALS AND METHODS

### Gut model.

Three triple-stage chemostat gut models were assembled to simulate CDI, as previously described (13). Briefly, three glass vessels were arranged in a weir cascade formation, maintained at 37°C, and pH controlled to represent the proximal (vessel 1, 5.5 ± 0.2), medial (vessel 2, 6.2 ± 0.2), and distal (vessel 3, 6.8 ± 0.2) human colonic environment (see Fig. S1). Vessel 1 has an operating volume of 280 ml, whereas vessels 2 and 3 operate at 300 ml, and each vessel simulates the nutrient availability and alkalinity observed in the area of the colon represented. An anaerobic environment is achieved by sparging the system with nitrogen, and a complex growth medium (13) connected to vessel 1 at a preestablished rate of 0.015h^−1^ ensures nourishment. The microbial abundance within the gut model has previously been validated against the intestinal contents of sudden-death victims, and it provides a close simulation of bacterial activities and composition in different areas of the colon (28). *In vivo* studies have shown the distal colon to be severely affected during human CDI (29); thus, in our model, vessel 3 is considered to be most physiologically relevant in terms of propensity to induce CDI. The gut model has been used to assess *in vitro* drug efficacy against simulated CDI at various stages of preclinical and clinical drug development, with data from *in vitro* models (14, 23) correlating well with data from animal models and phase 3 clinical trials (30–32).

### Experimental design.

**FIG 5:**
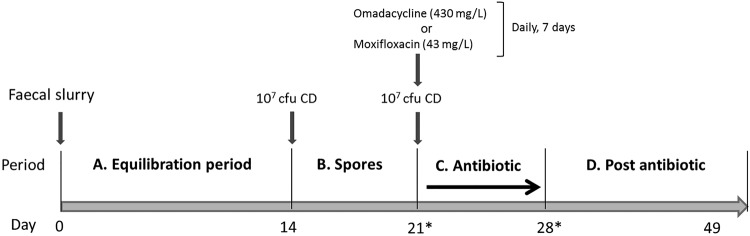
Outline of the gut model experiments with omadacycline (OMC1) and moxifloxacin (MOX). Asterisks indicate the period when model OMC1 diverged from MOX. CD, C. difficile spores.

A gut model experiment (OMC) was conducted to assess the effects of omadacycline in the intestinal microflora and in C. difficile populations. To confirm the initial observations, a second assay was later performed. The subsequent experimental design included a new gut model exposed to omadacycline (OMC1), run in parallel with a gut model dosed with moxifloxacin (MOX) (Fig. 5). Moxifloxacin is a broad-spectrum fluoroquinolone that has been shown to induce simulated CDI *in vivo* (1) and in the gut model (12). It was also the comparator antibiotic chosen for the omadacycline phase 3 clinical trial and, therefore, was selected as a positive control for the induction of simulated CDI in this study.

The OMC model was inoculated with fecal emulsion (10% wt/vol in prereduced PBS) prepared from C. difficile-negative feces of three healthy volunteers, while OMC1 and MOX models were inoculated with a fecal emulsion prepared from C. difficile-negative feces of five healthy volunteers. All donors were anonymous, ≥60 years of age, and with no history of antimicrobial therapy for 3 months.

Following inoculation with fecal emulsion, models were left without intervention for 2 weeks to reach a steady state (period A). A single 1-ml aliquot of spores (10^7^ CFU/ml) of C. difficile strain 210 (BI/NAP1/PCR ribotype 027/toxinotype III) (23) was inoculated into vessel 1 of each model (period B). Seven days later, at the start of antimicrobial dosing period, a second inoculum of spores (10^7^ CFU/ml) was added to vessel 1. A PCR ribotype 027 C. difficile strain was chosen for the study due to the clinical relevance of this ribotype. The 210 strain was initially isolated in 2005, during a CDI outbreak at the Maine Medical Centre (Portland, ME) and was kindly supplied by Robert Owens. Clinically relevant antimicrobial dosages were used during period C. Models OMC and OMC1 were dosed with 430 mg/liter of omadacycline (once daily, 7 days), while model MOX received 43 mg/liter of moxifloxacin (once daily, 7 days). Antimicrobial dosing was adjusted to achieve the desired concentration in the 280-ml volume of vessel 1. During a phase 1 clinical trial, ∼80% of a single oral radiolabeled dose (300 mg) of omadacycline was excreted in the feces (17). This equated to a maximum fecal omadacycline concentration of 423,000 ngEq/g or ∼430 mg/Kg (unpublished data). A dosing regimen of 430 mg/liter once daily was therefore used in this study, equating to 120 mg dosed daily into vessel 1 of the gut model.

The moxifloxacin dosing regimen was performed as previously described (12). Moxifloxacin concentration was based on published data of the concentration of this antimicrobial in human feces. Following the antimicrobial period, the models were monitored for a further 21 days with no interventions (period D).

### Enumeration of endogenous bacteria, and quantification of C. difficile toxin.

Gut microflora populations were monitored using viable counting on selective and nonselective agars, as described previously (33). Populations were measured in triplicate (three technical replicates of a single biological replicate) in vessels 2 and 3 every other day during period A and daily from period B onward. C. difficile total viable counts (TVC) and spore counts were also monitored daily in vessels 1, 2, and 3 throughout the experiment (periods A–D). Spore counts were obtained through serial dilution and plating of gut model fluid after alcohol shock. The C. difficile cytotoxin was monitored from period B onward using a quantitative Vero cell cytotoxicity assay (33). Cytotoxin titers were correlated to an arbitrary log_10_ scale and expressed as relative units (RUs) at the highest dilution, with >70% cell rounding (i.e., 10^0^, 1RU; 10^−1^, 2RUs; and 10^−2^, 3RUs). The limit of detection was ∼1.2 log_10_ CFU/ml for total counts, ∼1.5 log_10_ CFU/ml for spore counts, and 1 RU for toxin titer.

### Antimicrobial bioassay.

Antimicrobial concentrations in each gut model vessel during periods C and D were determined using a microbiological bioassay, as described previously (33). Concentrations of omadacycline were determined using Wilkins-Chalgren agar (Oxoid) with Kocuria rhizophila as the indicator organism. Concentrations of moxifloxacin were determined using Iso-Sensitest agar (Oxoid) with Escherichia coli as the indicator organism.

### Ethics statement.

The collection/use of fecal donations from healthy adult volunteers following informed consent was approved by the Leeds Institute of Health Sciences and Leeds Institute of Genetics, Health and Therapeutics and Leeds Institute of Molecular Medicine, University of Leeds joint ethics committee (reference HSLTLM/12/061).

## Supplementary Material

Supplemental file 1
