# Supplementary material for: Omadacycline Gut Microbiome Exposure Does Not Induce Clostridium difficile Proliferation or Toxin Production in a Model That Simulates the Proximal, Medial, and Distal Human Colon
Source: Antimicrob Agents Chemother. 2019 Jan 29;63(2):e01581-18. doi: 10.1128/AAC.01581-18 (PMC6355569; doi:10.1128/AAC.01581-18)
Supplement: Supplemental file 1 [file a86577ef4a44bb75840d427985ec5793_AAC.01581-18-s0001.pdf]

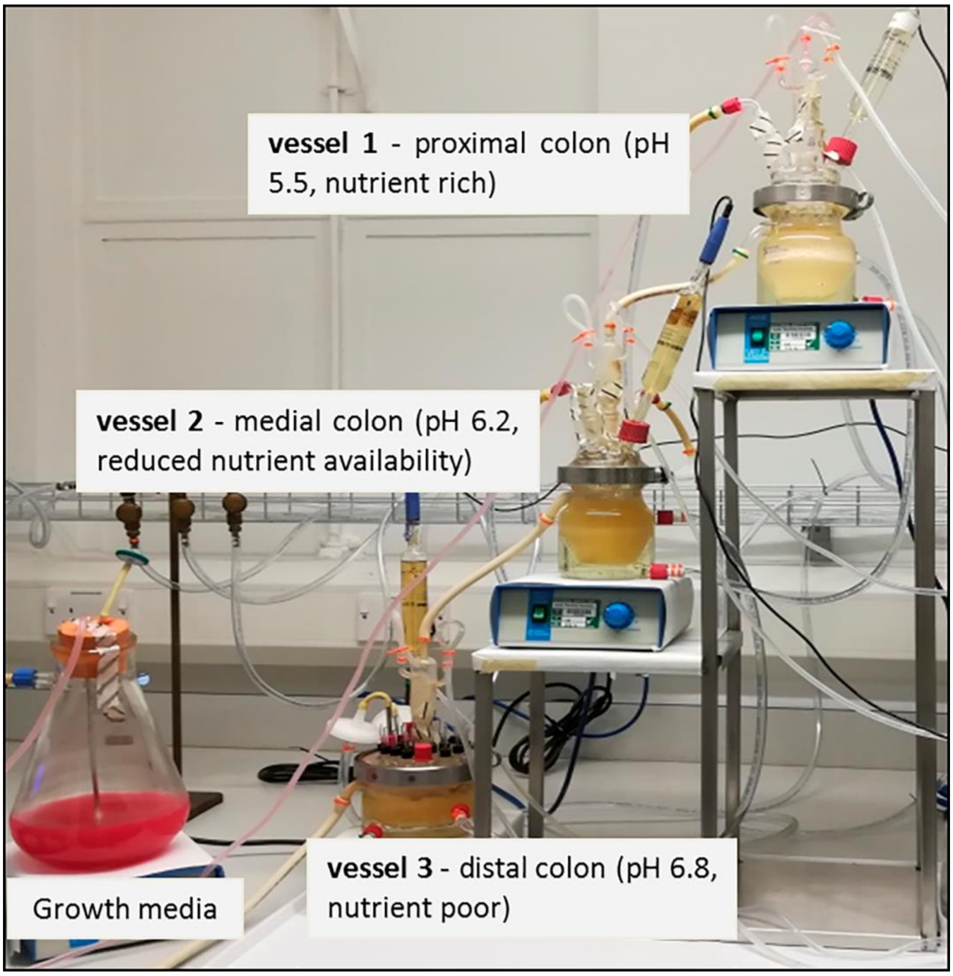

FIG S1 Representation of the triple stage *in vitro* gut model.

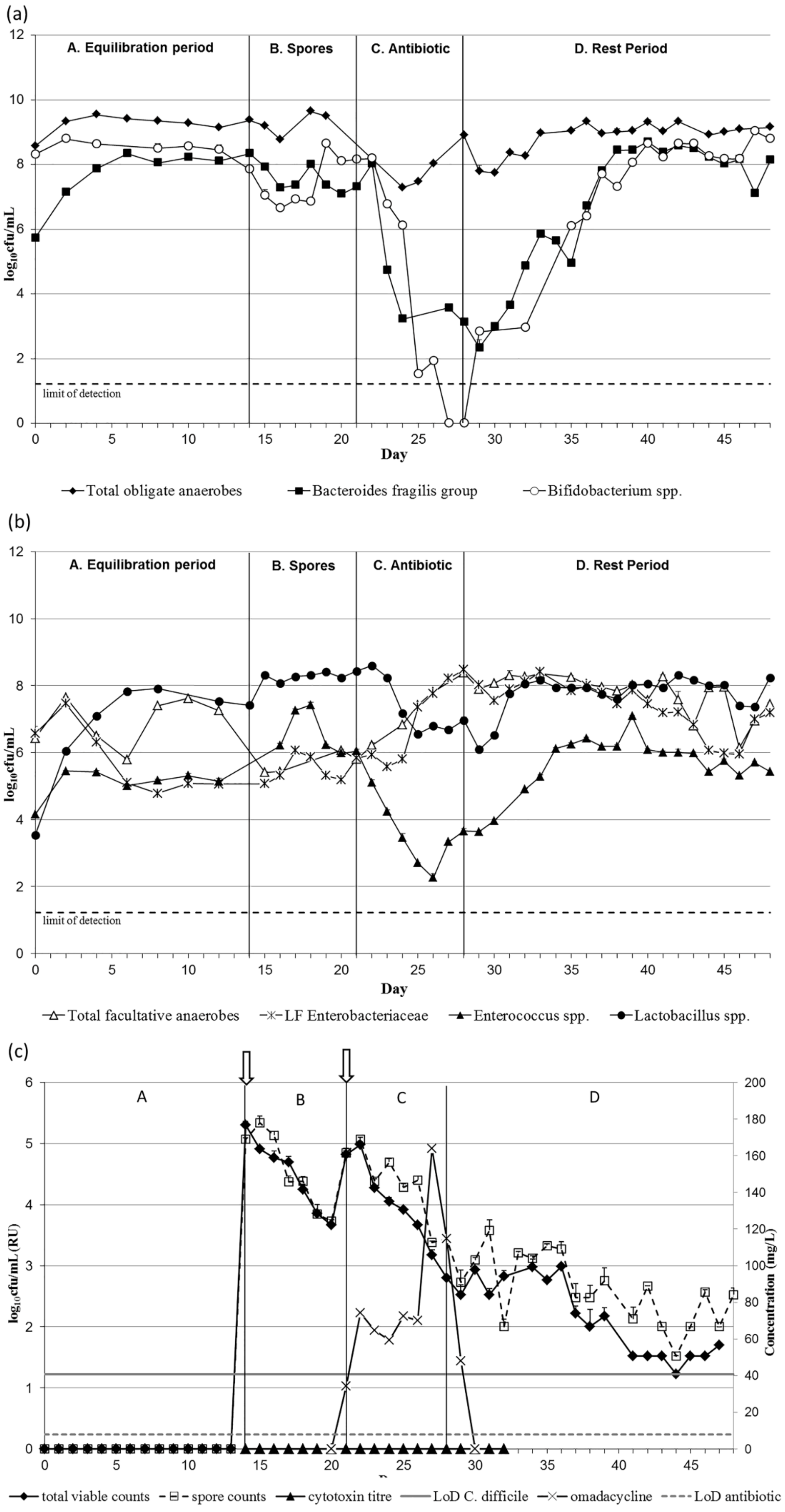
(a)
A. Equilibration period
B. Spores
C. Antibiotic
D. Rest Period
log10cfu/mL
limit of detection
Day
Total obligate anaerobes
Bacteroides fragilis group
Bifidobacterium spp.
(b)
A. Equilibration period
B. Spores
C. Antibiotic
D. Rest Period
log10cfu/mL
limit of detection
Day
Total facultative anaerobes
LF Enterobacteriaceae
Enterococcus spp.
Lactobacillus spp.
(c)
A
B
C
D
log10cfu/mL (RU)
Concentration (mg/L)
total viable counts
spore counts
cytotoxin titre
LoD C. difficile
omadacycline
LoD antibiotic

FIG S2 Mean (a) obligate anaerobic gut microbiota populations (log_{10} cfu/mL) including standard error bars, (b) facultative anaerobic gut microbiota populations (log_{10} cfu/mL), (c) *C. difficile* total viable counts and spore counts (log_{10} cfu/mL), cytotoxin titres (relative units, RU) and antimicrobial concentration (mg/L), in vessel 3 of model OMC. Vertical arrows mark the addition of *C. difficile* spores to the model, horizontal arrow marks the period of antibiotic instillation. LF *Enterobacteriaceae*, lactose-fermenting *Enterobacteriaceae*; LoD, limit of detection.

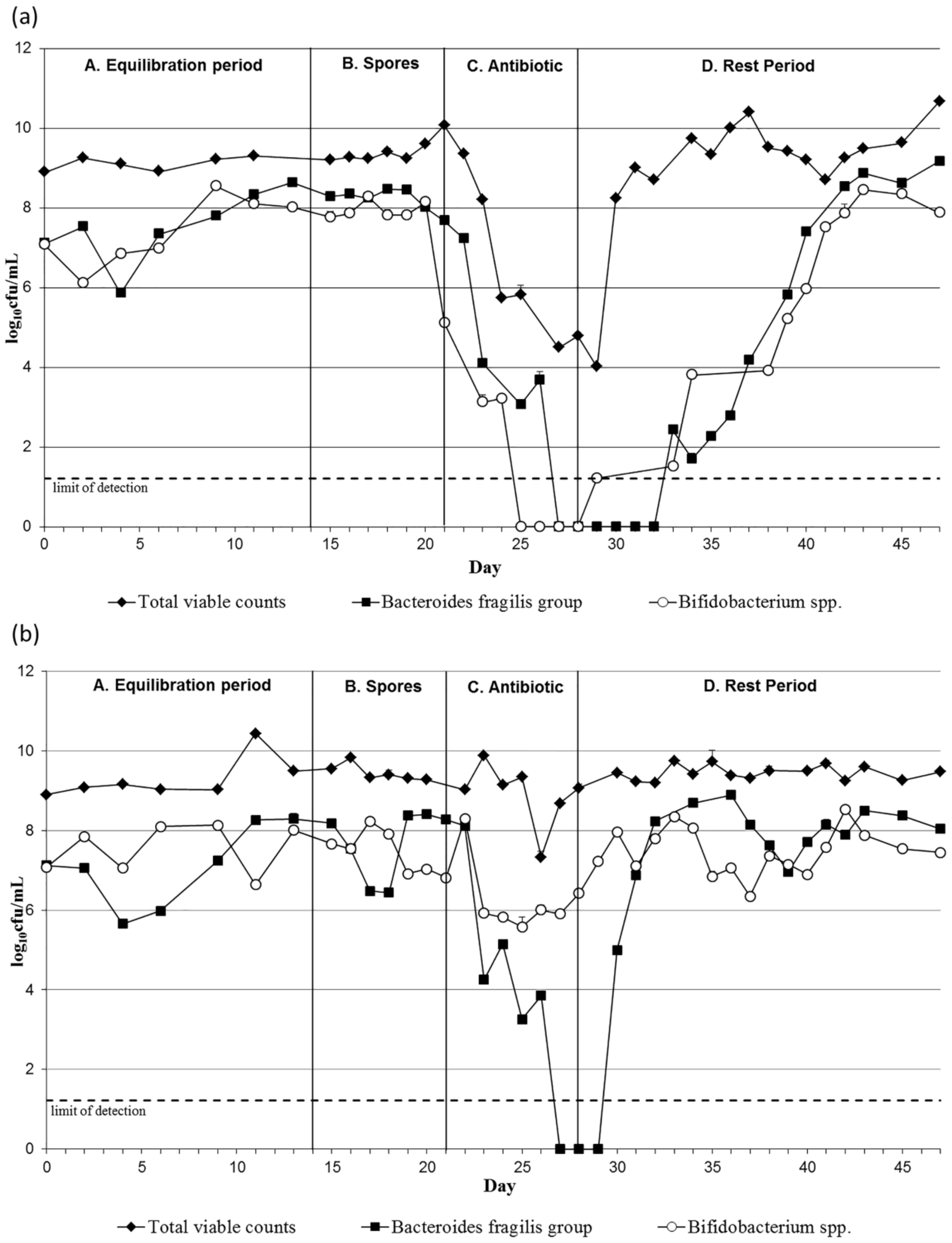

FIG S3 Mean obligate anaerobic gut microbiota populations (log_{10} cfu/mL) including standard error bars, in vessel 3 of (a) model OMC1 (omadacycline dosing), (b) model MOX (moxifloxacin dosing).

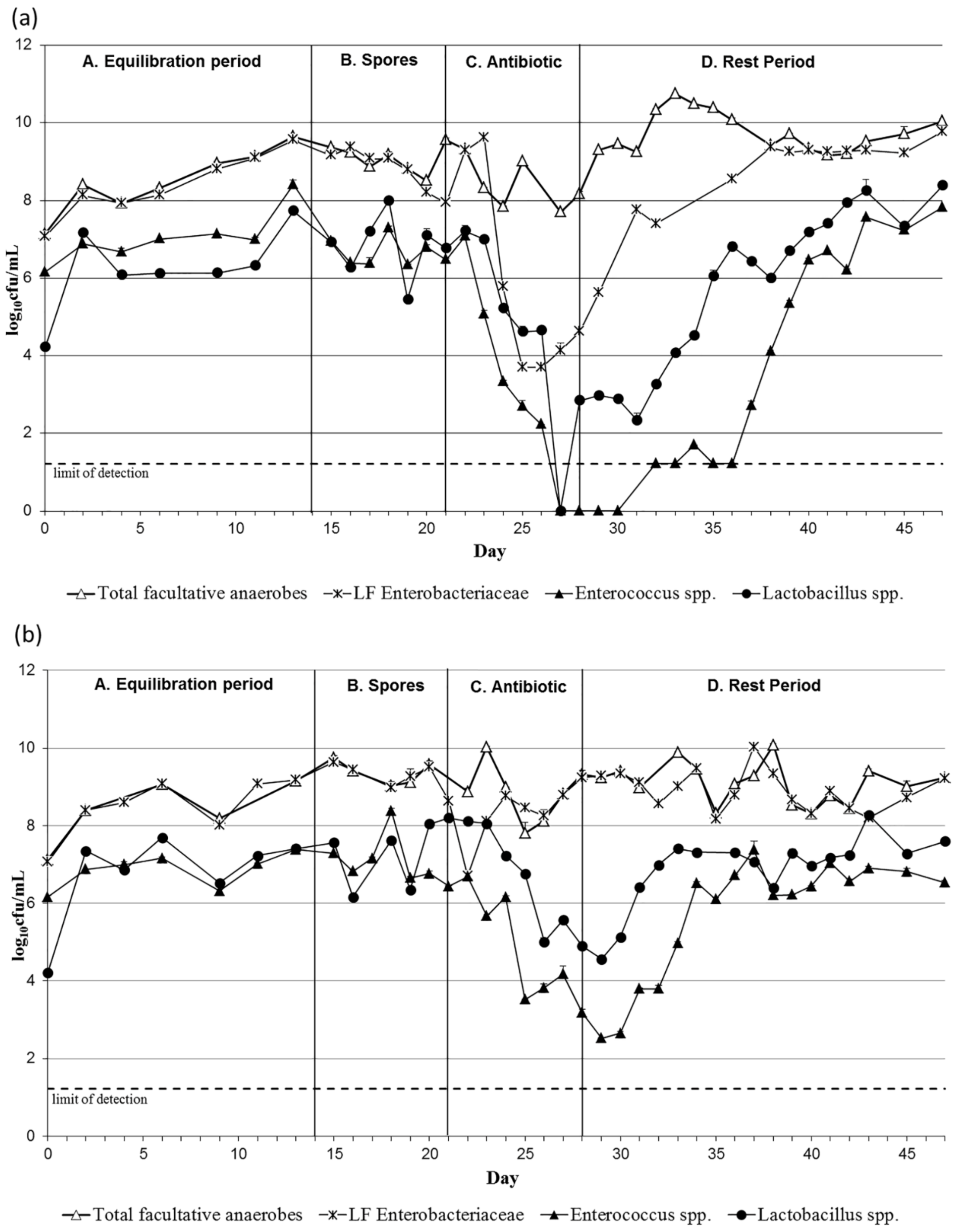

FIG S4 Mean facultative anaerobic gut microbiota populations ($\log_{10}$ cfu/mL) including standard error bars, in vessel 3 of (a) model OMC1 (omadacycline dosing), (b) model MOX (moxifloxacin dosing). LF *Enterobacteriaceae*, lactose-fermenting *Enterobacteriaceae*.

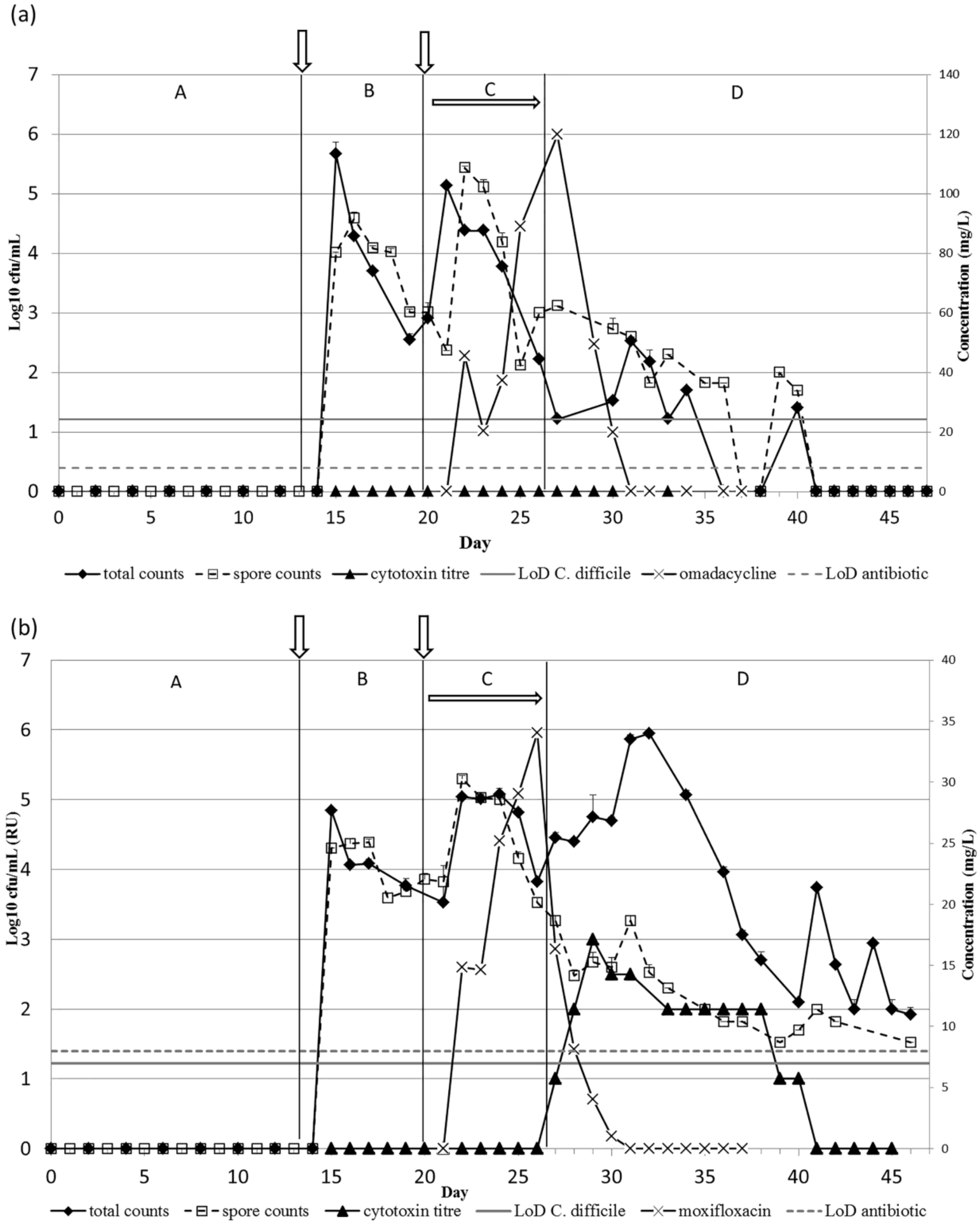

FIG S5 Mean *C. difficile* total viable counts and spore counts (log_{10} cfu/mL), cytotoxin titres (relative units, RU) and antimicrobial concentration (mg/L) in vessel 2 of (a) model OMC1 (omadacycline dosing), (b) model MOX (moxifloxacin dosing). Periods A–D are defined in Figure 5. Vertical arrows mark the addition of *C. difficile* spores to the model, horizontal arrow marks the period of antibiotic instillation. LoD, limit of detection.
